# Systemic immune‐inflammation landscape in brain metastasis needing neurosurgical resection: Analysis of 230 consecutive cases in a single center

**DOI:** 10.1002/iid3.694

**Published:** 2022-09-07

**Authors:** Jia‐Wei Wang, Ke Hu, Hai‐Peng Qian, Qing Yuan, Qi Liu, Chao Ma, Liujiazi Shao, Jing‐Hai Wan

**Affiliations:** ^1^ Department of Neurosurgery, National Cancer Center/National Clinical Research Center for Cancer/Cancer Hospital Chinese Academy of Medical Sciences and Peking Union Medical College Beijing People's Republic of China; ^2^ Department of Neurosurgery, The Third People's Hospital of Hefei Hefei Third Clinical College of Anhui Medical University Hefei People's Republic of China; ^3^ Department of Anesthesiology, Beijing Friendship Hospital Capital Medical University Beijing People's Republic of China

**Keywords:** brain metastasis, immune, inflammation, neurosurgical resection

## Abstract

**Background:**

Systemic immune‐inflammation states across the heterogeneous population of brain metastases are very important in the context of brain‐immune bidirectional communication, especially among the patients needing neurosurgical resection. Four blood cell ratios based on complete blood count (CBC) test serving as prognostic biomarkers have been highlighted by previous studies, including systemic immune‐inflammation index (SII), neutrophil‐to‐lymphocyte ratio (NLR), platelet‐to‐lymphocyte ratio (PLR), and lymphocyte‐to‐monocyte ratio (LMR). However, the presurgical systemic immune‐inflammation landscape in brain metastasis needing neurosurgical resection is limited.

**Methods:**

Patients with brain metastases admitted to the Department of Neurosurgery at the National Cancer Center, Cancer Hospital of Chinese Academy of Medical Sciences between January 2016 and December 2019 were included. Based on peripheral blood cell counts in CBC test before neurosurgical resection, four systemic immune‐inflammation biomarkers (SII, NLR, PLR, and LMR) were calculated. We characterized the changes of SII, NLR, PLR, and LMR in patients with brain metastasis before neurosurgical resection and the associations of these types of immune‐inflammation states with patient demographics. In parallel, the corresponding data from the relative healthy populations without systemic diseases were enrolled as the control in the present study.

**Results:**

Brain metastases induced systemic immune‐inflammation perturbation, which was characterized by a significant increase in SII (*p* < .01) and NLR levels (*p* < .01) and a significant decrease in the LMR level (*p* < .01) in comparison with the healthy control group. Moreover, patients with male gender, less Karnofsky Performance Status (KPS) scores (<70), specific pathological subtypes, extracranial transfer, and history of both systemic and radiation therapy may have significant differences in one or more of these biomarkers, which indicated poorer systemic immune‐inflammation states.

**Conclusions:**

This study provides evidence that brain metastasis is associated with perturbations in presurgical systemic immune‐inflammation states. We should pay attention to the systemic immune‐inflammation perturbations following brain metastasis in clinic, especially in the subpopulations with high risks.

## Graphical abstract

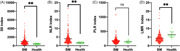

## 1 INTRODUCTION

As a leading cause of mortality and morbidity worldwide, brain metastasis has received considerable attention in the clinical and preclinical scenario.^1^, ^2^ Over the past decades, immune‐inflammation perturbation has been showed as the hallmark of peripheral cancer, which is associated with tumorigenesis, tumor growth, cancer progression, and patient survival.^3^, ^4^, ^5^, ^6^ To date, immune‐inflammation research in the cancer area has focused heavily on local immune responses in the tumor microenvironment (TME), systemic immune‐inflammation landscape beyond TME remains to be fully determined, especially in the patients with brain metastases needing neurosurgical resection.^6^, ^7^, ^8^, ^9^ Moreover, further progress toward the effective immunotherapeutic strategies requires a deeper understanding of the systemic immune‐inflammation relationships between tumors and their hosts across the body. Thus, an accurate assessment of systemic immune‐inflammation landscape across the heterogeneous population of brain metastases is very important, which may facilitate clinical decision‐making and appropriate stratification of future clinical trials.

Multiple molecules including various types of immune cells, blood cells, and other biochemical or hematological cells have been used to map the systemic immune‐inflammation landscape in clinic and labs.^5^, ^8^, ^10^ Considering the accessibility, convenience, and cost–benefit analysis among the assessing methods, biomarkers based on complete blood count (CBC) test are relatively promising in clinic because almost all the patients with brain metastases have the CBC test that is routinely measured in clinic.^8^, ^10^, ^11^, ^12^, ^13^, ^14^ Specifically, four blood cell ratios have been highlighted by previous studies, including systemic immune‐inflammation index (SII), neutrophil‐to‐lymphocyte ratio (NLR), platelet‐to‐lymphocyte ratio (PLR), and lymphocyte‐to‐monocyte ratio (LMR) based on peripheral neutrophil, lymphocyte, monocyte, and platelet counts. A vast of studies have indicated that these four blood cell ratios could serve as potential biomarkers for cancer incidence risk, early identification of disease, prognosis, and the response to immune checkpoint inhibitors in various human malignancies.^3^, ^4^, ^15^, ^16^, ^17^ To the best of our knowledge, the systemic immune‐inflammation landscape in brain metastasis needing neurosurgical resection based on CBC test is limited.

This study is aimed to fully characterize the types of presurgical immune‐inflammation states in brain metastasis needing neurosurgical resection and the associations of these types of immune‐inflammation states with patient demographics such as age, tumor origin, and treatment history to inform future therapeutic development and mechanistic studies in brain metastasis.

## 2 METHODS

This study was approved by the Ethics Committee of the Cancer Hospital, Chinese Academy of Medical Sciences (No.22/052‐3253) and was performed in accordance with the World Medical Association Declaration of Helsinki.

This was a retrospective study. Medical records from patients with brain metastases admitted to the Department of Neurosurgery at the National Cancer Center (NCC), Cancer Hospital of Chinese Academy of Medical Sciences between January 2016 and December 2019 were respectively reviewed. Brain metastasis was defined as intracranial parenchymal metastasis from peripheral cancer that was further confirmed by postoperative pathology. Skull metastases and leptomeningeal carcinoma (metastasis) were excluded from final analysis. All the included patients with brain metastasis in this study underwent neurosurgical tumor resection. And the surgery was indicated for the symptomatic, large, or accessible solitary lesions or in the circumstances there is a single large lesion that is life threatening or producing mass effect among multiple lesions in our center.

Data concerning the demographic parameters and presurgical CBC test results of each patient were retracted from the medical records, including age and gender of patients, Karnofsky Performance Status (KPS) scores, locations and numbers of brain metastases, extracranial transfer, pathology subtype, treatment information, and peripheral neutrophil, lymphocyte, monocyte, and platelet counts before neurosurgical resection. In parallel, the corresponding data from the healthy populations without systemic diseases (volunteers) were enrolled as the control in this study.

Based on peripheral blood cell counts in CBC test before neurosurgical resection, four systemic immune‐inflammation biomarkers (SII, NLR, PLR, and LMR) were calculated; Calculations were as follows, SII = (neutrophils × platelets)/lymphocytes, NLR = neutrophils/lymphocytes, PLR = platelets/lymphocytes, and LMR = lymphocytes/monocytes.

All statistical analyses and graphing were performed using SPSS Statistics software version 26 (SPSS Inc.) and Prism version 9.0 (GraphPad software). The data were presented as number or mean ± standard deviation. The unpaired *t* test, *χ* ^2^ test, or one‐way analysis of variance (ANOVA) test with least significant difference as a post‐hoc test were used to compare the intergroup difference. *p* value with less than .05 was considered statistically significant.

## 3 RESULTS

### 3.1 Brain metastases induced systemic immune‐inflammation perturbation

**Figure 1:**
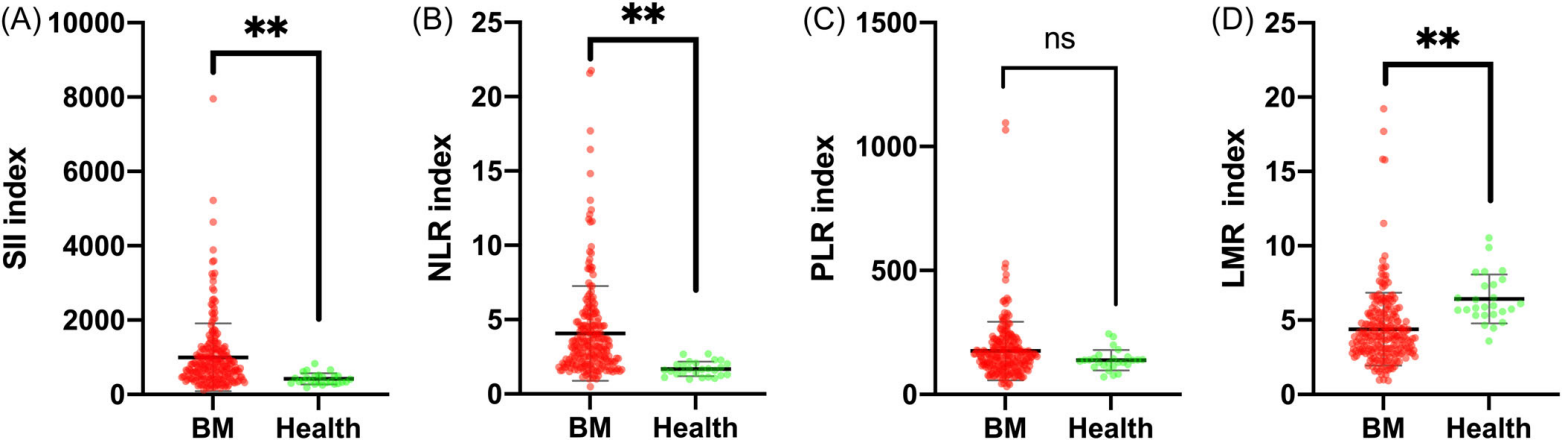
Brain metastases induced systemic immune‐inflammation perturbation. It was characterized by a significant increase in SII and NLR levels and a significant decrease in the LMR level in comparison with the healthy control group. BM, brain metastasis; LMR, lymphocyte‐to‐monocyte ratio; NLR, neutrophil‐to‐lymphocyte ratio; PLR, platelet‐to‐lymphocyte ratio; SII, systemic immune‐inflammation index. **p < .01,^ns^ *p* > .05.

In this study, 230 patients with brain metastases (age: 58.09 ± 10.15years) that were prepared for neurosurgical resection were enrolled into the study, with 127 males and 103 females (Table 1). In addition, 26 relatively healthy individuals without systemic diseases (age: 53.42 ± 6.01 years) were studied as the control group for comparison (13 males and 13 females). There were no significant differences regarding age and gender between brain metastases and healthy group (*p* > .05). As shown in Figure 1, brain metastases induced systemic immune‐inflammation perturbation, which was characterized by a significant increase in SII(*p* < .01) and NLR levels (*p* < .01) and a significant decrease in the LMR level (*p* < .01) in comparison with the healthy control group.

**Table 1:** Clinicopathological parameters in 230 patients with brain metastases prepared for neurosurgical resection

Variables	Brain metastases
Age (years)	58.09 ± 10.15
Gender	
Male	127
Female	103
KPS scores	91.78 ± 16.37
Primary lesions	
Lung	169
Breast	20
Kidney	10
Colorectum	7
Melanoma	4
others	20
Diagnostic order of primary and metastatic lesions	
Primary lesion firstly diagnosed	113
BM firstly diagnosed	117
BM locations	
Supra. dominant	179
Infra. dominant	51
BM number	
Single	123
Multiple	107
Extracranial transfer	
No	164
Yes	66
History of systemic therapy^a^ or radiotherapy^b^	
Naïve	127
Systemic therapy only	52
Radiotherapy only	11
Both systemic or radiotherapy	40

*Note*: Data were presented as number or mean ± standard deviation.

Abbreviations: BM, brain metastasis; Infra., infratentorial; KPS, Karnofsky Performance Status; Supra., supratentorial.

^a^ Including chemotherapy, molecularly targeted therapy and immunotherapy.

^b^ Including stereotactic radiosurgery and whole brain radiation therapy.

### 3.2 Systemic immune‐inflammation states and patient demographics

**Figure 2:**
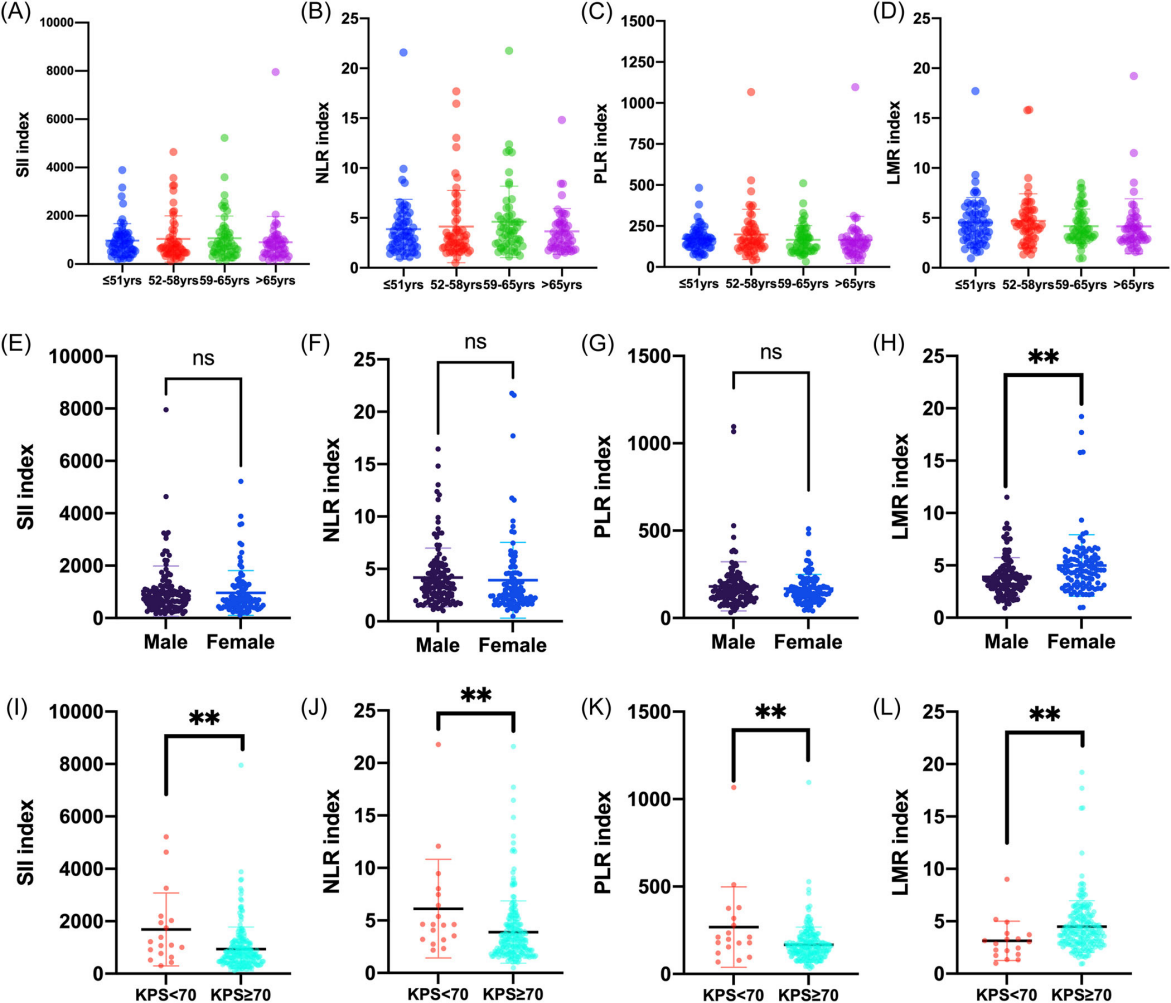
Associations of immune‐inflammation biomarkers with age, gender, and KPS scores. Data showed patients with KPS scores greater than or equal to 70 had remarkable decrease of SII, NLR, and PLR levels, and increase of LMR index (I–L). BM, brain metastasis; KPS, Karnofsky Performance Status; LMR, lymphocyte‐to‐monocyte ratio; NLR, neutrophil‐to‐lymphocyte ratio; PLR, platelet‐to‐lymphocyte ratio; SII, systemic immune‐inflammation index. **p < .01,^ns^ *p* > .05.

To explore the associations of immune‐inflammation states with the patient demographics, SII, NLR, PLR, and LMR levels in brain metastases were further analyzed according to the demographical categories. Our study indicated that there were no significant differences in SII, NLR, PLR, and LMR levels between groups with different ages (*p* > .05) (Figure 2A–D). In addition, female patients showed significantly increased LMR index in comparison with male patients (Figure 2E–H). Patients with KPS scores greater than or equal to 70 had a remarkable decrease of SII, NLR, and PLR levels (*p* < .01), and an increase of LMR index (*p* < .01) (Figure 2I–L).

**Figure 3:**
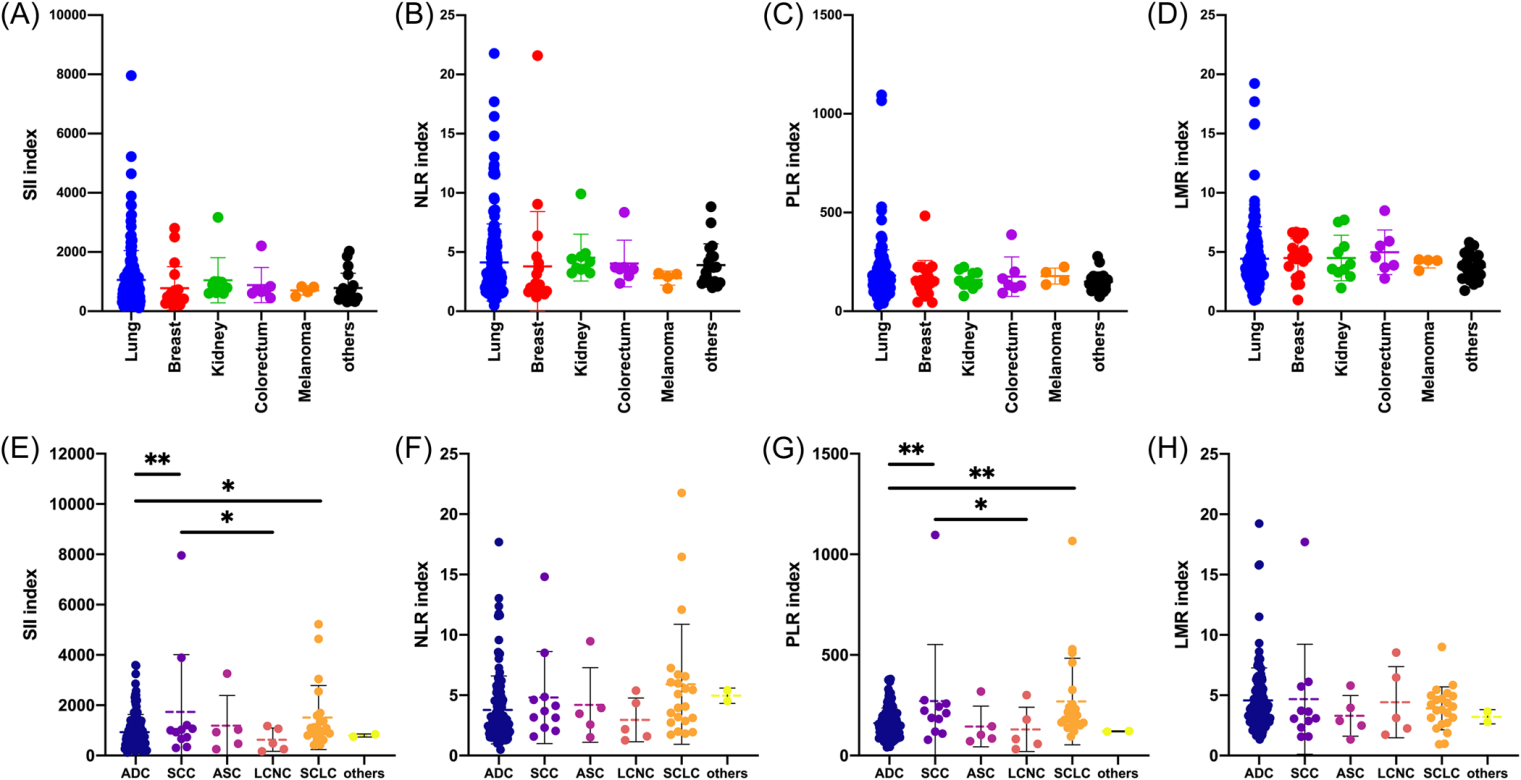
Associations of immune‐inflammation biomarkers with primary sites and pathological subtypes. There were no significant differences in SII, NLR, PLR, and LMR levels among patients with different primary sites (A–D). Furthermore, our data showed the SII and PLR levels were significantly increased in patients with SCC and SCLC in comparison with the one with ADC (E–H). ASC, adenosquamous carcinoma; ADC, adenocarcinoma; LCNC, large‐cell neuroendocrine carcinoma; LMR, lymphocyte‐to‐monocyte ratio; NLR, neutrophil‐to‐lymphocyte ratio; PLR, platelet‐to‐lymphocyte ratio; SII, systemic immune‐inflammation index, SCC, squamous cell carcinoma; SCLC, small cell lung cancer. *p < .05, **p < .01.

In this study, the most common primary site for brain metastasis was lung, accounting for 73.48% of the total patients, which was following by breast (8.70%), kidney (4.35%), colorectum (3.04%), and melanoma (1.74%) (Table 1). As shown in the Figure 3A–D, there were no significant differences in SII, NLR, PLR, and LMR levels among patients with different primary sites (*p* > .05). The pathological subtypes of patients with lung cancer brain metastasis included adenocarcinoma (ADC, *n* = 124), squamous cell carcinoma (SCC, *n* = 11), adenosquamous carcinoma (ASC, *n* = 5), large‐cell neuroendocrine carcinoma (LCNC, *n* = 5), small cell lung cancer (SCLC, *n* = 22), and others (*n* = 2). Furthermore, we explored the immune‐inflammation states in subtypes of patients with lung cancer. Our data showed the SII and PLR levels were significantly increased in patients with SCC and SCLC in comparison with the one with ADC (Figure 3E–H). In addition, patients with SCC also showed significant differences in SII and PLR levels compared with LCNC (*p* < .05).

**Figure 4:**
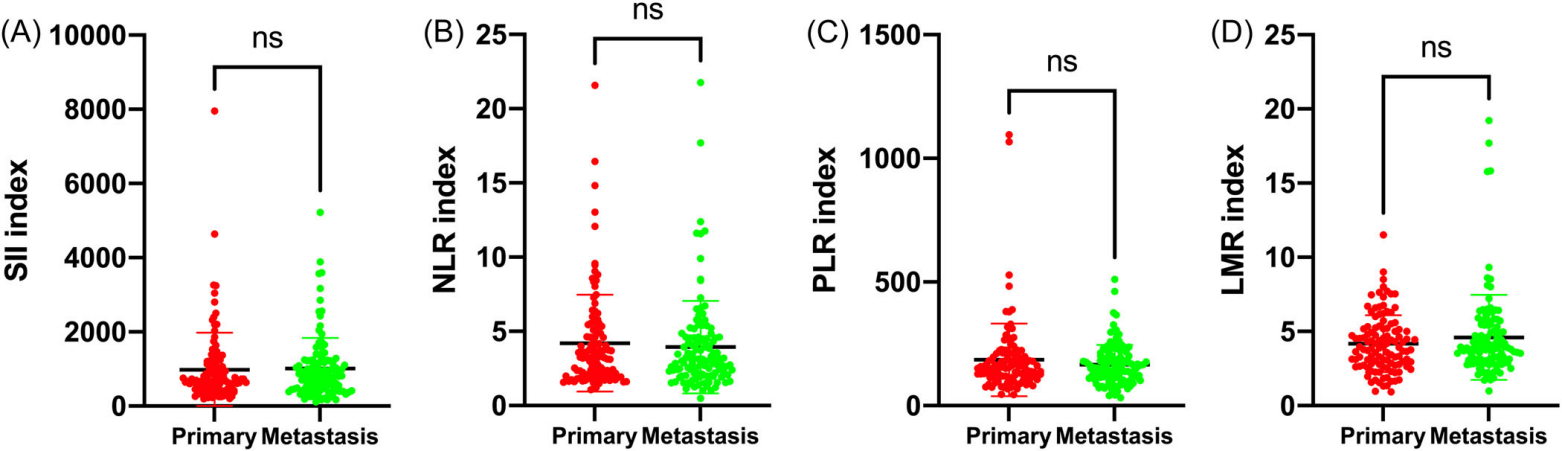
Associations of immune‐inflammation biomarkers with diagnosis sequence of brain metastasis. Brain metastases were categorized into two types, one(group primary) was primary lesion firstly diagnosed and brain metastasis subsequently diagnosed, the other(group metastasis) was brain metastasis firstly diagnosed and then primary lesion diagnosed. LMR, lymphocyte‐to‐monocyte ratio; NLR, neutrophil‐to‐lymphocyte ratio; PLR, platelet‐to‐lymphocyte ratio; SII, systemic immune‐inflammation index. ^ns^ *p* > .05.

According to the diagnostic sequence of primary and metastatic lesions, the patients with brain metastases admitted to our institution could be categorized into two types. One type was that brain metastasis presented major symptoms and was initially diagnosed before primary lesion (*n* = 117), the other type was that brain metastasis occurred after the diagnosis and treatment of primary lesions (*n* = 113) (Table 1). Our data indicated that there were no significant differences in SII, NLR, PLR, and LMR levels between the two types of brain metastases (*p* > .05) (Figure 4A–D).

**Figure 5:**
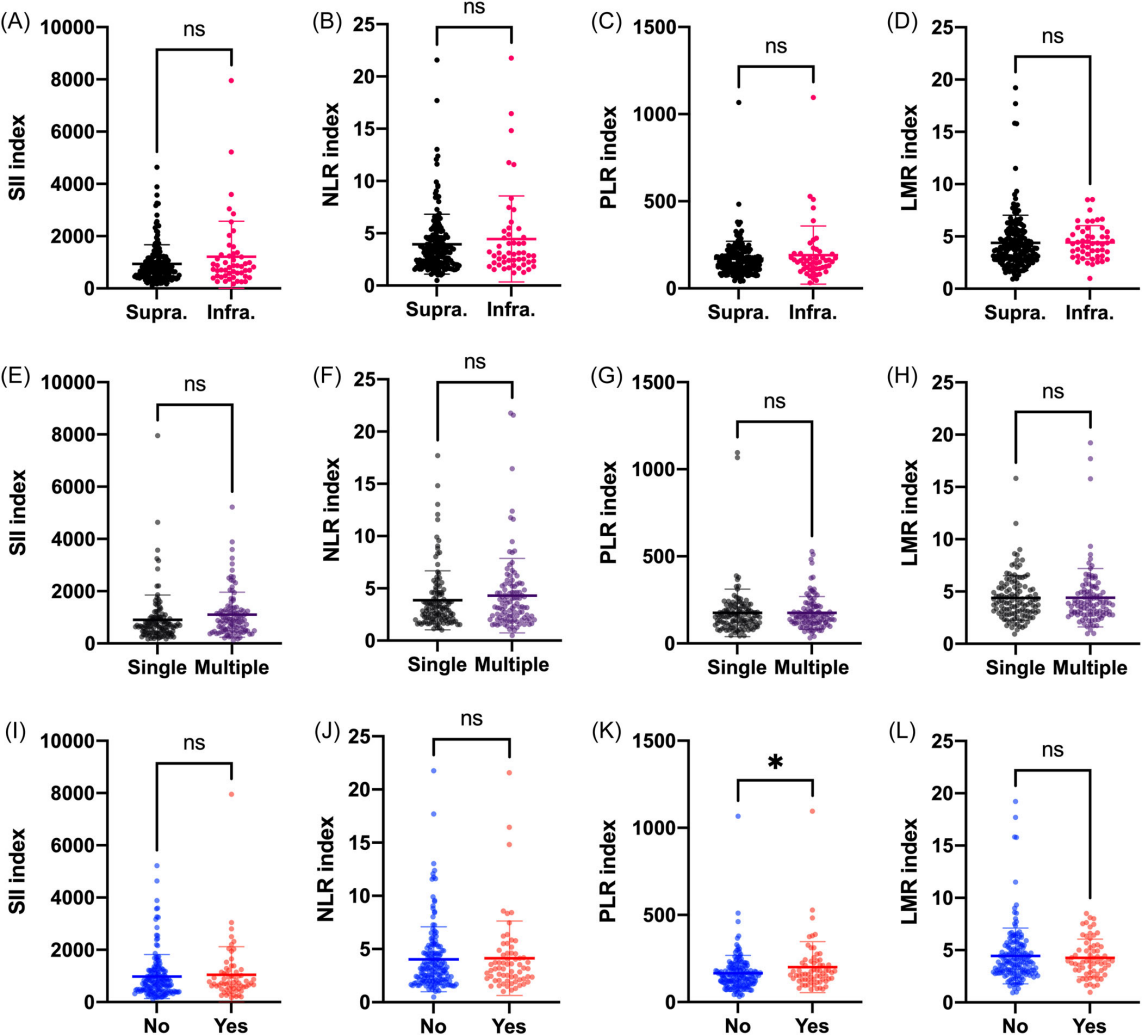
Associations of immune‐inflammation biomarkers with locations and number of brain metastasis and extracranial transfer state. Our data indicated that the patients with extracranial transfer had significantly increased PLR level (K). LMR, lymphocyte‐to‐monocyte ratio; NLR, neutrophil‐to‐lymphocyte ratio; PLR, platelet‐to‐lymphocyte ratio; SII, systemic immune‐inflammation index. ^ns^ *p* > .05, *p < .05.

**Figure 6:**
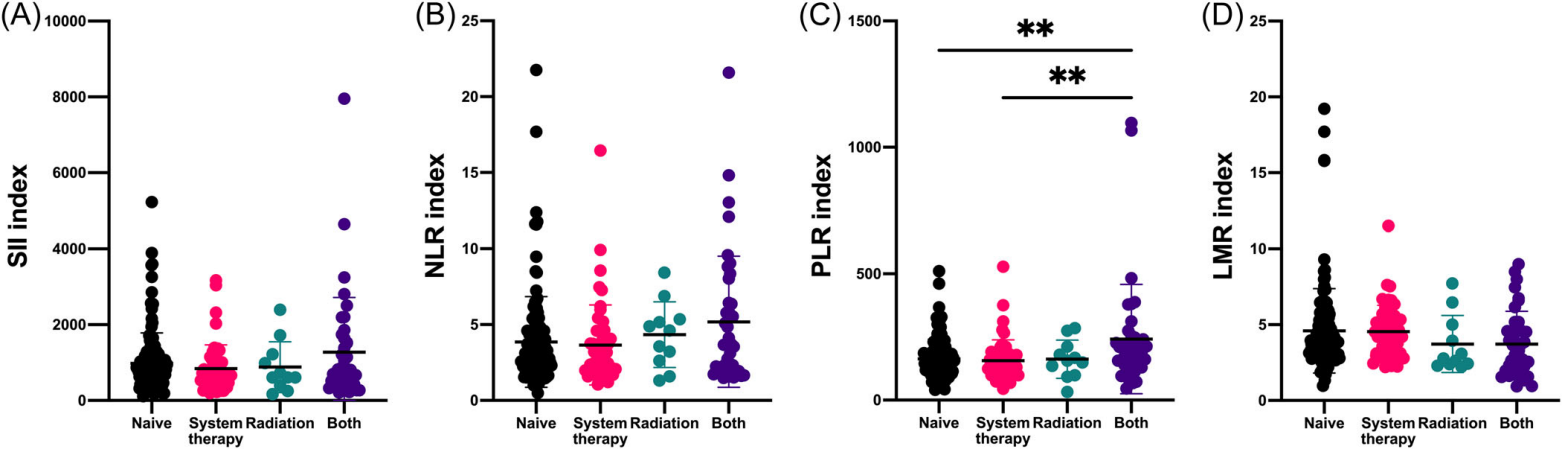
Associations of immune‐inflammation biomarkers with treatment history of brain metastasis. Four types of treatment history for brain metastasis before neurosurgical resection except for surgery of primary sits included treatment‐naïve, systemic therapy, radiation, and both systemic therapy and radiation. We found the patients with both systemic therapy and radiation had significantly increased PLR level in comparison with the one with treatment‐naïve or systemic therapy. LMR, lymphocyte‐to‐monocyte ratio; NLR, neutrophil‐to‐lymphocyte ratio; PLR, platelet‐to‐lymphocyte ratio; SII, systemic immune‐inflammation index. **p < .01.

As shown in Figure 5, our data found that there were no significant differences in SII, NLR, PLR, and LMR levels regardless of the locations and number of brain metastases (*p* > .05) (Figure 5A–H). Figure 5K indicated that the patients with extracranial transfer had significantly increased PLR level (*p* < .05). Table 1 summarized the four types of treatment history for brain metastasis before neurosurgical resection except for surgery of primary sites, including treatment‐naïve, systemic therapy, radiation, and both systemic therapy and radiation. We found the patients with both systemic therapy and radiation had increased SII, NLR, and PLR levels and decreased LMR level in comparison with the one with other treatment history while the difference was significant in PLR level (*p* < .01)(Figure 6).

## 4 DISCUSSION

In this study, four biomarkers (SII, NLR, PLR, and LMR) based on the presurgical peripheral blood cell count were used to depict the systemic immune‐inflammation states in the specific population of brain metastases who needed neurosurgical resection and to stratify the patients with high risks of impaired systemic immune‐inflammation states. The main findings are as follows: (1) brain metastasis can induce significant perturbations in systemic immune‐inflammation states characterized by an increase in SII and NLR levels and a decrease in the LMR levels. (2) Patients with male gender, less KPS scores (<70), specific pathological subtypes, extracranial transfer, and history of both systemic and radiation therapy may have poorer systemic immune‐inflammation states. These findings remind us that we should pay attention to the systemic immune‐inflammation perturbations following brain metastasis, especially in some subpopulations of patients with high risks.

It is well‐known that complex interaction of multiple mechanisms may be attributed to the final impaired systemic immune‐inflammation states following brain metastasis. First of all, the intense bidirectional communication between the central nervous system ^18^ and immune system has been highlighted during the past decades, which mainly involves three pathways of neuroimmunomodulation: the hypothalamus–pituitary–adrenal (HPA) axis, the sympathetic nervous system (SNS) and the parasympathetic nervous system.^5^, ^19^, ^20^ Previous clinical and experimental evidence have shown that CNS injury, such as stroke,^21^ traumatic brain injury,^22^ and spinal cord injury,^23^ can induce a disturbance of the normally well‐balanced interplay between the immune system and the CNS, and eventually leads to brain‐specific secondary immunodeficiency that is nominated as CNS injury‐induced immunodepression syndrome (CIDS) by Christian Meisel and his coauthors.^19^ Moreover, based on three distinct models of brain cancer, including GL261 glioma, B16 melanoma, and in a spontaneous model of diffuse intrinsic pontine glioma, Katayoun Ayasoufi and his colleagues recently have also demonstrated that the immunosuppression is not unique to brain cancer itself, but rather occurs in response to brain injury,^24^ which is consistent with our findings. In the present brain metastasis case series, neurosurgical resection is indicated especially when there is a symptomatic, large, or accessible solitary lesion or in the circumstances there is a single large lesion that is life‐threatening or producing mass effect among multiple lesions. Obviously, mass effects resulting from the intra‐axial metastatic lesions and peritumoral edema can lead to significant CNS injury and subsequently induce CIDS.

Treatment strategies for cancer are considered as another key factor in the modulation of systemic immune‐inflammation states.^6^, ^8^, ^25^, ^26^ In the present case series, the treatment before admission to our department mainly includes three mainstays: surgery/biopsy of primary lesions, systemic antitumor therapy such as chemotherapy, molecularly targeted therapies and immunotherapies, and radiation therapy. And further analysis indicated that 107 patients (91.45%) in group of BM firstly diagnosed did not have any treatment while only 20 patients (17.70%) in the group of primary lesion firstly diagnosed received single surgery/biopsy of primary lesions and did not have systemic antitumor therapy or radiation therapy (Table 1). As shown in Table 1 and Figure 6, our study provides evidence that systemic antitumor therapy or radiation therapy may influence the systemic immune‐inflammation states and the significant perturbation in systemic immune‐inflammation states occurs in the one with both systemic antitumor therapy and radiation therapy. In terms of the association between systemic immune‐inflammation states and treatment strategies for cancer in this extremely heterogeneous population of patients who develop brain metastases, several points should be kept in mind. For example, primary tumor burden can induce extensive disruption of hematopoiesis across multiple immune organs, which is characterized by the expansion of immature neutrophils and monocytes in the periphery.^6^, ^27^, ^28^ However, surgical resection of primary tumor is also likely followed by both detrimental (such as immunosuppression early after surgical procedures) and beneficial effects (such as reduced primary tumor burden) on the systemic immune‐inflammation states.^29^, ^30^, ^31^, ^32^ Moreover, the remodeling of systemic immune by systemic antitumor therapy and radiation have highly depended on context, including intervention timing, dosing, combinations, cancer type, and stage.^25^, ^33^ Taken together, the dual character and comprehensive interaction of these treatment models may explain why the two types of brain metastasis with different treatment strategies before admission to our department did not have significant differences in systemic immune‐inflammation biomarkers.

A vast clinical studies about prognosis in brain metastasis have indicated that patients with high KPS scores, origin from lung ADC, or without extracranial transfer may have a favorable outcome in comparison with others, and these parameters have already been incorporated into prognosis graded scales for brain metastasis.^34^, ^35^, ^36^ In addition, previous studies have also shown negative correlation of SII, NLR, and PLR levels, and positive correlation of LMR levels with outcome in multiple cancer context.^3^, ^6^, ^10^, ^11^, ^13^, ^17^ This study showed that patients with high KPS scores, origin from lung ADC, or without extracranial transfer had better systemic immune‐inflammation states characterized by a decrease of SII, NLR, and PLR levels, and an increase of LMR level, which is consistent with previous studies. In terms of the association between gender and systemic immune‐inflammation states in cancer, our findings showed males were accompanied with poor systemic immune‐inflammation states, which are similar to most of previous studies involving non‐SCLC,^37^ nasopharyngeal carcinoma,^38^ and thyroid carcinoma.^39^ In addition, there are also some studies indicating there is no difference between males and females regarding to these biomarkers in gastric cancer.^40^

It should be noted that there are some limitations in this study. First, in this study, four biomarkers are analyzed to depict the systemic immune‐inflammation states, our findings cannot conclude one biomarker is superior to the other biomarkers although the changes of these four biomarkers are not synchronously significant in terms of the association between these biomarkers and patient demographics. Second, the sample of brain metastases from lung cancer accounts for 73.48% of the total patients and these results are further analyzed based on the pathological subtypes of lung cancer in our study. However, the sample of brain metastases from other primary lesions is relative small so we cannot make further analysis according to the molecular types. Finally, this study is a single‐center retrospective cohort study, and findings should be confirmed in other larger prospective cohorts.

In conclusion, this study provides evidence that brain metastasis is associated with perturbations in systemic immune‐inflammation states. Thus, we should pay attention to the systemic immune‐inflammation perturbations following brain metastasis in clinic, especially in the subpopulations with high risks including male gender, less KPS scores, specific pathological subtypes, extracranial transfer, and history of both systemic and radiation therapy.

## AUTHOR CONTRIBUTIONS

Qi Liu, Chao Ma, Liujiazi Shao, and Jing‐Hai Wan conceived and designed the study. Jia‐Wei Wang, Ke Hu, Hai‐Peng Qian, Qing Yuan, Liujiazi Shao, and Qi Liu performed the data collection and statistical analyses. Jia‐Wei Wang drafted the initial manuscript. Qi Liu, Chao Ma, Liujiazi Shao, and Jing‐Hai Wan made critical comments and revision for the initial manuscript. All authors reviewed and approved the final manuscript.

## CONFLICT OF INTEREST

The authors declare no conflict of interest.

## ETHICS STATEMENT

This study was approved by the Ethics Committee of the Cancer Hospital, Chinese Academy of Medical Sciences (No.22/052‐3253). All methods in this study were carried out in accordance with relevant guidelines and regulations.

## Data Availability

The datasets generated and/or analyzed during the current study are available from the corresponding author on reasonable request.
